# Population Data Centre Profile: The Manitoba Centre for Health Policy

**DOI:** 10.23889/ijpds.v4i2.1131

**Published:** 2020-02-25

**Authors:** A Katz, J Enns, M Smith, C Burchill, K Turner, D Towns

**Affiliations:** 1 Manitoba Centre for Health Policy, Rady Faculty of Health Sciences, University of Manitoba, 408-727 McDermot Ave, Winnipeg, Manitoba, Canada R3E 3P5

## Abstract

**Objective:**

To profile the Manitoba Centre for Health Policy (MCHP), a population health data centre located at the University of Manitoba in Winnipeg, Canada.

**Approach:**

We describe how MCHP was established and funded, and how it continues to operate based on a foundation of trust and respect between researchers at the University of Manitoba and stakeholders in the Manitoba Government’s Department of Health. MCHP’s research priorities are jointly determined by its scientists’ own research interests and by questions put forward from Manitoba government ministries. Data governance, data privacy, data linkage processes and data access are discussed in detail. We also provide three illustrative examples of the MCHP Data Repository in action, demonstrating how studies using a variety of Repository datasets have had an impact on health and social policies and programs in Manitoba.

**Discussion:**

MCHP has experienced tremendous growth over the last three decades. We discuss emerging research directions as the capacity for innovation at MCHP continues to expand, including a focus on natural language processing and other applications of artificial intelligence techniques, a leadership role in the new SPOR Canadian Data Platform, and a foray into social policy evaluation and analysis. With these and other exciting opportunities on the horizon, the future at MCHP looks exceptionally bright.

## Background

### A Brief History of the Manitoba Centre for Health Policy

The Manitoba Centre for Health Policy (**MCHP**) is a population health data centre located at the University of Manitoba^1^ in Winnipeg, Canada. It was formally established in 1991, but the story of how it came to be extends back two more decades, when the two researchers who would later become its co-founders first arrived in Manitoba [1].

In 1973, Les and Noralou Roos were working in the business school at the University of Manitoba, having recently completed doctoral degrees in political science at the Massachusetts Institute of Technology. The Dean of Medicine, having decided that aligning the faculty’s research programs with the new universal health care system was an important priority, arranged a meeting between the Rooses and the lead physician at the Manitoba Health Services Commission. The province’s practice of routinely recording hospital and physician contacts for the whole population of Manitoba opened the door to new research opportunities using anonymized provincial health data [1]. These early studies [2,3] were funded by external agencies based on topics of interest to the Department of Manitoba Health, Seniors & Active Living (shortened here to ‘Manitoba Health’) and clinical experts, and resulted in collaborations with researchers based at Dartmouth College in the U.S.

As their grant funding and publication records grew, Les and Noralou Roos gained national and international recognition for their work. The Canadian Institute for Advanced Research, which was focusing on the role of socioeconomic status and education as health determinants, convinced the Minister of Health that investing in the Roos research capabilities made sense; a three-year contract to establish and launch the Manitoba Centre for Health Policy and Evaluation (as it was then called) was signed in 1991 [1]. A Canadian Foundation for Innovation (**CFI**) grant received in 1999 was used to fund new office space and to bring the Department of Education and the Department of Family Services datasets into MCHP. In 2001, MCHP was awarded the Health Services Research Advancement Award by the Canadian Health Services Research Foundation.

Since 1991, the funding model has been a contract between the provincial government and MCHP for research ‘deliverables’. The focus for these projects is determined by interests of and issues facing the Department of Health in periodic meetings between MCHP and the Deputy Minister of Health. In addition, many MCHP researchers and affiliated scientists hold faculty positions at the University of Manitoba [4] and lead independent research programs supported by research grants from the Canadian Tri-Council agencies and other funding bodies.

## Approach

### The Manitoba Centre for Health Policy: Governance and Research Priorities

The MCHP Director is appointed by the Department of Community Health Sciences, and reports through the Department Head to the Dean of the College of Medicine at the University of Manitoba. The Director leads an executive team comprising four Associate Directors (in the areas of Research, Repository and Deliverables, Data Access and Use, and Planning and Development), whose collective role is to guide MCHP in achieving its strategic priorities [5]. MCHP also operates under the guidance of an advisory board comprising both government and university appointees [6]. The board currently includes deputy ministers from government departments, senior university administrators and public representatives.

MCHP has had an ongoing contract with the Manitoba Government’s Department of Health for completing arm’s-length research projects since 1991. Proposed project topics are submitted by MCHP researchers, from within government departments, and by the provincial health region leads. Five topics are chosen annually by Manitoba Health, including at least one project on child health selected by the Healthy Child Committee of Cabinet. MCHP employs approximately 60 staff, including researchers, data analysts, research project coordinators, and support staff.

### Operating Model

MCHP acts as the steward of data routinely collected during the delivery of public services in Manitoba. The data are derived primarily from Manitoba Government departments, including Manitoba Health, Manitoba Families, Manitoba Education & Training, Manitoba Justice, and several other cross-departmental government agencies. Other data come from provincial laboratories, clinical programs, community and social outreach organizations, and Indigenous governance bodies. The 90+ datasets are held in the centralized Manitoba Population Research Data Repository (shortened here to ‘the Repository’) at MCHP. See **Supplementary File 1** for a sample of Repository datasets.

### Population & Setting

The Repository contains information on nearly all residents of the province of Manitoba, with coverage for some datasets extending as far back as 1970 [7]. Manitoba is a central Canadian province with a universal healthcare system and a population of about 1.3M people [8]. About 57% of the population resides in the major urban centre, Winnipeg [9]. The remainder live in rural settings, mainly in the southern part of the province. The age distribution of Manitoba is similar to the rest of Canada, with residents age 0-14 making up 19%, age 15-64 making up 65%, and age 65+ making up the remaining 16% of the total population [8].

While the Repository data cover the vast majority of Manitoba residents, a few groups are not well represented. Military personnel and individuals incarcerated in federal prisons are federally insured and are therefore not part of the provincial health insurance registry. This means that although they are included in the Repository, their records are incomplete in select datasets. Indigenous populations in Manitoba (First Nations, Metis, and Inuit peoples) may not be completely identified in some datasets, as some data are not collected in reserve communities.

### Data Governance & Legislation

The Manitoba Government and other data providers listed in the section above are the trustees of the data in the Repository. As the custodian of this sensitive information, MCHP adheres to the rules for privacy and protection of personal information outlined in the province’s Personal Health Information Act (**PHIA**) [10] and the Freedom of Information and Protection of Privacy Act (**FIPPA**) [11] of Manitoba. Depending on the data source and use, requirements for data disclosure and use at MCHP might also be influenced by the Mental Health Act of Manitoba, [12] the Youth Criminal Justice Act of Canada, [13] the Child and Family Services Act of Manitoba, [14] the Statistics Act of Canada, [15] and the Privacy Act of Canada, [16] and others.

### Consent Model

Ensuring the privacy and confidentiality of individual Manitoban’s personal information is a priority for MCHP. In Manitoba, PHIA grants individuals the right to access to their own medical records, and the right to privacy, which ensures that their personal health information will be protected from unauthorized collection, use, disclosure, retention and destruction [10]. PHIA upholds these rights by placing limits on how trustees can handle a person’s medical records. Meanwhile, FIPPA provides individuals the right to access information in records (other than health records) held by public bodies [11]. Under this provincial legislation, individual patients or participants must give consent for disclosure and use of their data in research when direct contact with these individuals is anticipated.

However, for projects involving MCHP Repository data, the Manitoba Health Information Privacy Committee (**HIPC**) and the University of Manitoba Health Research Ethics Review Board (**UM-HREB**) typically waive this requirement for individual consent, for several reasons: first, research projects using the Repository data are a secondary use of data, and therefore there is no direct contact with patients or participants; second, the ‘greater good’ of the research outweighs the risk of intrusion on Manitobans’ privacy, and it is impractical to obtain consent; and third, measures (such as de-identification and limited access to the Repository data) have been taken to protect individual privacy. While it is technically possible to 'opt out’ of some Manitoba Health administrative data systems, fewer than 0.1% of individuals have ever done so. Privacy measures at MCHP are described in more detail in the sections below.

### Data Linkage

MCHP uses a trusted third-party system to ensure that newly acquired datasets and data updates being brought into the Repository have been stripped of any directly identifying information [17]. Briefly, the data provider sends any **demographic data** that could be used to identify an individual (e.g., names, address, phone numbers) and an internal reference number to the Information Management and Analytics Unit of Manitoba Health. There, the identifying information is used to match each individual to their existing 9-digit Personal Health Identification Number (**PHIN**) using a custom-developed software package called LINKPRO. The process starts with deterministic approach, with probabilistic follow-up on non-matched individuals; several passes through the data using different combinations of variables helps to ensure this match is as certain as possible. Next, the directly identifying details are removed from the records, and a scrambled (encrypted) version of the PHIN is attached. The scrambled PHIN is generated using a consistent, standard algorithm and is permanently stored in each record. The number is scrambled the same way for each individual’s records. This ensures that the records can be linked together at a later date, but protects the person’s identity.

At the same time that the data are being de-identified by Manitoba Health, the data provider also sends the internal reference numbers and **program data** (e.g., clinical test results or other public service use information) to MCHP. The de-identified records are sent electronically to MCHP and kept separate from the Repository until approvals from the data provider are in place. The program data are linked to the scrambled PHINs by the internal reference number. The data within the Repository are then linked on a project-by-project basis using the scrambled PHIN.

It is important to note that at no point in the data linkage process does any party have possession of all of the pieces of the linkage puzzle: the data provider does not have access to the scrambled PHIN, Manitoba Health does not have access to the program data, and MCHP does not have access to the identifying information. This system safeguards the privacy and security of the data. This third party approach has been very successful in building trust between partners, allowing the work that MCHP does to proceed with confidence. 

### Data Architecture & Information Technology

The Repository data are stored in a SAS-based SQL server providing user and project level access controls. Analytic systems are supported on Microsoft Windows 2012 servers, providing ETL/Acquisition, MCHP internal analytics, and remote access within Manitoba (as stipulated by the agreement between Manitoba Health and MCHP). Remote access is supported from Microsoft Windows-based computers and requires unique individual accounts with two factor authentication assigned by MCHP. The platform supports SAS as the default analytic environment, although STATA and R software are also available.

### Data Quality

MCHP follows a data quality framework providing a comprehensive and consistent evaluation of every database and update received [17,18]. Assessing data quality is particularly important for secondary use of information, as MCHP rarely has control over the data collection and maintenance processes. The data quality framework was developed from a review of quality assessment practices in other Canadian and international population data centres, and includes as core components the concepts of accuracy (e.g., completeness, correctness), internal and external validity, timeliness and interpretability.

### Privacy by Design

Several layers of protections are in place to ensure data privacy for individuals whose information is included in the Repository. These privacy measures include:

- All research projects must be reviewed for privacy, ethics, and impact by individual data providers, HIPC and UM-HREB to ensure the data are being used appropriately.
- Before accessing the Repository, individuals with access to an MCHP computer system and all principal investigators on MCHP projects complete an accreditation session, which provides an overview of the data access and use process. They are required to sign a pledge of confidentiality and an agreement that the analyses will be conducted in alignment with MCHP’s research processes and the Government of Manitoba’s requirements.
- System and data access are tracked, and individuals using the analytic systems at MCHP must log in using their unique ‘userID’ with two-factor authentication.
- Data access is provided to individual users based on the level of permissions they have obtained for their research projects. Access is provided only to individuals who have been identified as having ‘line level access’ on approved projects.
- Data extracted for non-MCHP researchers or analysts is the minimum level required to complete the outlined research project(s).
- The Repository contains no directly identifying information such as names or addresses.
- Information taken off of the MCHP analytic systems must be aggregate or statistical in nature with no strata representing an identifiable individual. This is implemented by requiring all aggregate data to represent at least 6 individuals or events, and any suppressed information (1-5 individuals or events) cannot be recreated through the use of simple math. Information taken off of the analytic system is manually reviewed for project association and small numbers.
- Prior to presenting or publishing data, the material must be reviewed by the data providers to ensure appropriate use within the scope of the project, flag any chance of re-identifying individuals, and confirm suitable representation of associated programs or departments. This review must be conducted at least 10 days prior to presentations and 30 days prior to publication.

### Data Access and Publication

Access to Repository data must follow the requirements established by MCHP and individual data providers. This process is outlined on the MCHP website [19], and described briefly below. Research project set-up steps include:

1. Complete MCHP Accreditation Session.
2. Submit MCHP Feasibility Request (estimate letter and approvals required by the MCHP Data Access Unit).
3. Obtain approvals from UM-HREB, HIPC and data providers; requires proof of funding for the research project.
4. Complete a signed Research Agreement between the data provider and the researcher or research institution.
5. Initiate data system set-up and data extraction, and review project requirements with MCHP analyst.
6. If remote access to the Repository is required, an MCHP computer account will need to be configured.

#### Timeline

Completing steps 1-4 typically takes an average of 3-4 months, but this can vary considerably depending on whether the research is privately or publically funded, whether the researcher has sought approvals before requesting a feasibility review, and whether the request is well-defined. The time required for step 5 depends on the complexity of the project (e.g., number of datasets and fields to be extracted), but with as many as 20 analysts available at MCHP, completing this step usually takes days or weeks (not months). Step 6 can be completed in a day or two.

#### Other Considerations

Access to datasets must occur through MCHP-based systems. Remote access from physically secure locations and with appropriate approvals can be arranged. Researchers preparing to publish findings using the Repository data must submit a draft of the publication ahead of public release to the data providers for review and feedback. The final version of a presentation or publication must be provided to HIPC once the presentation is complete or the publication is accepted.

### Noteworthy Outputs

In this section, we describe three studies that used data from a variety of Repository sources to make an impact on health and social policies and programs in Manitoba.

#### (1) Enhancing Academic Programming for Students from Low-Income Families

Our report on Manitoba children’s educational outcomes included an analysis of Grade 12 standardized test scores by socioeconomic status [20]. The left-hand panel of Figure 1 illustrates a mild income gradient, with students from the poorest families having an average passing rate of 75% on the test, while students from the highest income areas had an average passing rate of 95%. However, this panel only shows results for students who wrote the test. The right-hand panel shows the results for both students who wrote the test and students who should have written the test had they progressed through the school system as expected. This latter group includes students who were held back a year or dropped out of school. The income gradient in this population-based analysis is much steeper, with passing rates at only 16% for the lowest income students and 80% for the highest income students.

**Performance of Grade 12 Students in Manitoba by Socioeconomic Status:**
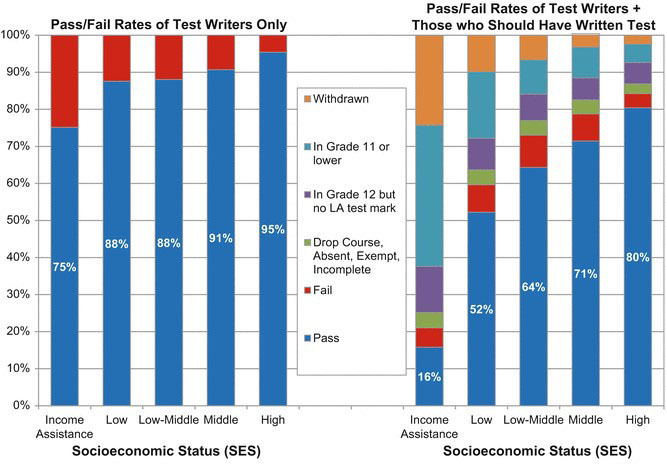
Versions of this figure are in Brownell et al (2004) and Roos et al (2010) – used with permission

Brownell M, Roos NP, Fransoo R, et al. How Do Educational Outcomes Vary with Socioeconomic Status? Key Findings from the Manitoba Child Health Atlas 2004. Winnipeg, Canada: Manitoba Centre for Health Policy; 2004.

Roos NP, Roos LL, Brownell M, Fuller EL. Enhancing policymakers’ understanding of disparties: relevant data from an information-rich environment. 2010 Milbank Q. 88(3):382-403.

These findings, together with other analyses presented in our 2001 Child Health Atlas [21], led to the development of the *Community School Investigators (CSI)* program [22], which provides services to enrich and enhance the academic achievement of children in inner-city low-income neighbourhoods.

#### (2) Establishing the Positive Impacts of an Unconditional Prenatal Benefit

In 2001, Manitoba introduced the Healthy Baby Prenatal Benefit (HBPB) to improve prenatal health and birth outcomes in families with a documented annual income of less than CAN $32,000. The program provides eligible women with an unconditional income supplement of up to $81.41/month during their second and third trimester of pregnancy. In addition, pamphlets with information about prenatal and postnatal care (e.g., the importance of prenatal nutrition and information about breastfeeding) are included with the monthly payment [23].

In 2010, MCHP evaluated the impacts of the HBPB on the birth outcomes of recipients [24]. Using a quasi-experimental retrospective cohort design, we examined all births (2003-2010) to Manitoba women who were receiving income assistance and either did or did not receive the HBPB. The two groups had comparable low mean annual incomes at $9,941 and $9,972, respectively. Thus, for women who received the HBPB, the benefit represented an increase in their monthly incomes of almost 10%.

Remarkably, receiving the HBPB was significantly associated with a reduction in low birth weight births and preterm births, and an increase in breastfeeding initiation. Population-preventable fractions for low birth weight and pre term births were decreases of 21% and 17.5%, respectively, and the population-attributable fraction for breastfeeding was a 4% increase. These findings were subsequently published in *Pediatrics* [25], and together with a follow-up study demonstrating that receipt of HBPB was associated with increased population-level health equity [26], attracted the attention of news media in Canada [27] and the US [28,29]. This evidence of the effectiveness of the HBPB has also contributed to its longevity (nearly 20 years) as a Manitoba government program.

#### (3) Projecting Personal Care Home Bed Needs in Manitoba

Modern industrial societies are facing new challenges due to an increasing older adult population. Both the ‘baby boom’ and improved health and longevity of older populations have contributed to this phenomenon. In 2011, an MCHP report pointed to dramatic increases in the number of individuals over age 65 in Manitoba; this number is projected to rise from 14% of the population in 2009 to more than 18% in 2036 [30]. This creates potential challenges for governments planning for the needs of older adults in supportive housing, nursing homes (known as *personal care homes* in Manitoba), and long-term care facilities.

In 2012, MCHP released a second report examining to what extent Manitoba’s need for personal care home (PCH) beds, or alternatives such as supportive housing and extended home care, was expected to grow over the next several decades [31]. Figure 2 shows the actual and projected population numbers for three age groups: age 65-74 (top line), age 75-84 (middle line), and age 85+ (lower line). Note how the slope of each line changes at a different rate as the baby boom generation grows older.

**Actual and Projected Number of Older Adults Living in Manitoba, by Age Group:**
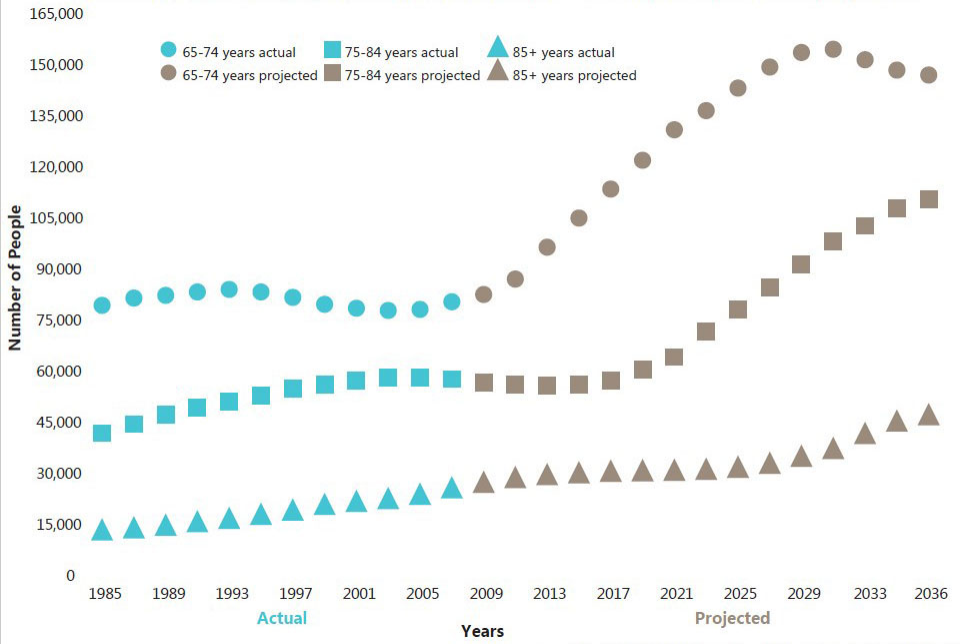
Figure from Chateau et al. (2012) – used with permission. Population projections calculated by Manitoba Bureau of Statistics (2008).

Chateau D, Doupe M, Walld R, Soodeen R-A, Ouelette C, Rajotte L. Projecting Personal Care Home Bed Equivalent Needs in Manitoba Through 2036. Winnipeg, MB; 2012

Manitoba Bureau of Statistics. Manitoba Regional Health Authorities Population and Demographic Projections 2006 to 2036. Winnipeg, MB: Government of Manitoba; 2008.

The PCH bed projection study generated a number of important findings. First, the proportion of older adults using PCHs has been shrinking since 1985. This trend was attributed to a healthier population of older adults and increased use of supportive housing and home care services. Second, since most PCH residents are age 85 or older, when baby boomers reach the age of 85 (starting in 2031), we would expect the number of days Manitobans spend in PCH facilities to rise dramatically. The study predicted that between 5,100 and 6,300 additional beds will be needed in personal care homes, supportive housing or expanded home care services – an increase of 55-70% more than current capacity. Third, researchers found that older adults who were married or had children were less likely to be residents of personal care homes. The findings of this report and related work by MCHP continue to serve as the basis for long-term healthcare planning by the Manitoba government and other agencies [32,33].

## Discussion

Since its inception, MCHP has been one of the central drivers of data linkage science and population health research in Canada and worldwide. The Manitoba Population Research Data Repository was initially known as the Population Health Data Repository. As the data centre has grown from maintaining 12 health service delivery datasets in 1991 to more than 90 datasets in 2019, the breadth of non-healthcare data led to the change to a more inclusive name. And while these data serve as a tremendous resource for researchers, clinicians and decision makers to better understand the Manitoba population’s health and social well-being, there are still many opportunities for growth and advancement.

For example, while many of the 90+ datasets in the Repository are regularly updated, the time lag from when the data are collected to when they are transferred to the Repository sometimes hinders researchers from providing up-to-date answers to time-sensitive questions. MCHP is dependent on data providers to make regular updates to datasets available, and negotiating more timely access to these records is part of ongoing discussions with Manitoba Health in regards to the impact of MCHP’s research deliverables.

There is work underway to acquire more clinical data into the Repository. Over the past several years, clinician-led projects have increased significantly but relatively few clinicians have recognized the potential the Repository holds to answer their research questions. MCHP continues to emphasize the value-add of linking clinical data to administrative health and social data, which together can provide greater context to clinical queries and bring innovative solutions into focus.

MCHP continues to grow our capacity to access and understand complex ‘free-text’ or ‘unstructured’ data in existing Repository datasets, such as clinician notes in electronic medical records, and case notes from social services files. These free-text data often contain unique types of identifiers, and entail additional processes for de-identification. Analyses of free-text require sophisticated language processing methods that have not yet become widely available in the health sector [34]. However, MCHP is currently working to adapt and apply machine-learning techniques and deep neural learning approaches used in the field of computer science, with the ultimate goal of addressing knowledge gaps and challenges in the health system and social sector.

MCHP continues to develop and build an innovative knowledge translation strategy that will integrate knowledge users into our research process and ensure that our research is policy-relevant. This strategy is based on MCHP’s long history of integrated knowledge exchange, including the work of a national award-winning research collaborative called the Need to Know Team [35–37], which facilitates knowledge sharing among university-based researchers, high-level planners from the health regions in Manitoba, and policy-makers from Manitoba Health. Established in 2001, this team has revolutionized how administrative data sources can be used to inform health and social policy issues.

Finally, MCHP recognizes that many of our strengths in advancing our work are the product of the fruitful partnerships formed with stakeholders, community organizations and decision makers. Our long-standing relationship with Manitoba Health has for many years been the foundation of MCHP and the research we do. More recently, we have formed partnerships with Indigenous groups in Manitoba through our work on health equity [38], primary care [39], and two soon-to-be-released reports on First Nations health status and access to healthcare. As well, MCHP plays a key role in the recently-announced Strategy for Patient-Oriented Research (SPOR) Canadian Data Platform, a national initiative to harmonize administrative health data across the provinces and territories [40].

### Lessons Learned

Throughout MCHP’s rich history, the many lessons we learned have proved invaluable for building and maintaining an enduring research enterprise. First, we have learned that building trust with stakeholders is absolutely essential to maintaining and expanding the Repository. Our commitment to ensuring that the Repository data and secure are de-identified means that the possibility of individuals in our studies being identified is extremely remote. This instills confidence amongst our stakeholders, and smooths the way for further interactions.

Second, we have come to appreciate how important it is to work closely with our partners throughout the research process (from beginning to end) to ensure we are interpreting their data correctly, and that we all understand both the context and limitations that accompany any data collection processes and analyses. Providing our partners a first look at results allows them to prepare for any possible policy implications that might arise. Typically, our agreements stipulate that data providers receive a 60-day review period prior to any research findings being publically released. However, this review does not allow for suppression or reinterpretation of findings unless gross errors have been made.

Third, we realized early on that keeping track of research methodology (how we measure things) was going to be critical for constancy of interpretation over time. In response, we developed the Concept Dictionary and Glossary, available to the public on MCHP’s website. The Concept Dictionary has been a vital resource over the years, not only in enhancing consistency in how we interpret our findings, but also in reducing the time and effort required to complete subsequent research projects.

Finally, whenever possible, we take the research interests of the data providers into account when we initiate new projects. In many cases, they serve as project co-investigators, allowing them to enhance their research acumen, provide important contextual information regarding the data or the findings, and initiate the knowledge translation process. It remains essential, however, that the principal investigator retains the right to report any findings that arise, and that the partnership agreement is supported by the rights and freedoms granted to academic researchers working at a Canadian university. 

## Conclusions

MCHP continues to lead in developing innovative research resources based on routinely collected administrative data, forging new techniques in cross-jurisdictional analysis, and building lasting relationships with policy makers and Indigenous partners. The announcement of substantial new funding in the form of the SPOR Canadian Data Platform promises ongoing opportunities to continue our trail-blazing role in using administrative data to answer policy-relevant questions. Healthcare delivery is evolving rapidly as new technology enters the clinical environment and system planners endeavor to bend the cost curve, making the availability of data to support health system change even more critical than before. These data need to be amenable to new Artificial Intelligence techniques and the data architecture able to support the greater computing power necessary to exploit these opportunities.

The combination of real-time clinical data and administrative claims data has huge appeal, due to the synergy between these different data types and the potential for capitalizing on the new knowledge they can generate. While our capacity to achieve this goal is still to be realized, planning of future hardware and data architecture requirements should include consideration of these future developments. To date, the dramatic improvements in computing power that will facilitate these types of change have not been matched with advances in privacy protection, and other challenges in benefiting from these advances still exist.

MCHP has been a leader in acquiring and using social data in analyses that incorporate and take into account the social determinants of health. Although the Repository has not yet contributed to social research in a significant way, new funding from the Social Sciences & Humanities Research Council has been used to establish a multi-sector partnership to conduct Social Policy Evaluation Collaborative Team Research at the Universities in Manitoba (**SPECTRUM**)^2^. Through SPECTRUM, MCHP is conducting applied research and evaluation on existing social services, programs and policies in Manitoba, melding the expertise of knowledge creators, mobilizers and users to address real-world policy questions through use of the Repository at MCHP.

With these and other exciting opportunities on the horizon, the future at MCHP looks exceptionally bright.

## Acknowledgments

Many thanks to Les Roos, Noralou Roos and Marni Brownell for their careful review of this paper.

## Ethics Statement

This work did not require ethical approval as it was descriptive and not a research study involving human participants.

## Footnotes

1 For more information on the Manitoba Centre for Health Policy, visit our homepage at http://umanitoba.ca/faculties/health_sciences/medicine/units/chs/departmental_units/mchp/

2 More information on SPECTRUM is available at www.spectrum-mb.ca.

## Abbreviations

CFI	Canadian Foundation for Innovation
CSI	Community Schools Investigators
FIPPA	Freedom of Information and Protection of Privacy Act
HBPB	Healthy Baby Prenatal Benefit
HIPC	Health Information Privacy Committee
MCHP	Manitoba Centre for Health Policy
PCH	personal care home (nursing home)
PHIA	Personal Health Information Act
PHIN	Personal Health Information Number
SPECTRUM	Social Policy Evaluation Collaborative Team Research at Universities in Manitoba
SPOR	Strategy for Patient-Oriented Research
UM-HREB	University of Manitoba Health Ethics Review Board
